# Efficacy of FimA antibody and clindamycin in silkworm larvae stimulated with *Porphyromonas gulae*

**DOI:** 10.1080/20002297.2021.1914499

**Published:** 2021-04-25

**Authors:** Sho Yoshida, Hiroaki Inaba, Ryota Nomura, Masaru Murakami, Hidemi Yasuda, Kazuhiko Nakano, Michiyo Matsumoto-Nakano

**Affiliations:** a Department of Pediatric Dentistry, Okayama University Graduate School of Medicine, Dentistry and Pharmaceutical Sciences, Okayama, Japan; b Department of Pediatric Dentistry, Osaka University Graduate School of Dentistry, Osaka, Japan; c Departments of Pharmacology, Veterinary Public Health II and Molecular Biology, School of Veterinary Medicine, Azabu University, Kanagawa, Japan; d Yasuda Veterinary Clinic, Tokyo, Japan

**Keywords:** Fimbriae, genotypes, *Porphyromonas gulae*, silkworm larvae

## Abstract

**Objective**: *Porphyromonas gulae*, a major periodontal pathogen in animals, possesses fimbriae that have been classified into three genotypes (A, B, C) based on the diversity of *fimA* genes encoding fimbrillin protein (FimA). *P. gulae* strains with type C fimbriae were previously shown to be more virulent than other types. In this study, we further examined the host toxicity mediated by *P. gulae* fimbriae by constructing recombinant FimA (rFimA) expression vectors for each genotype and raised antibodies to the purified proteins.

**Methods and Results**: All larvae died within 204 h following infection with *P. gulae* type C at the low-dose infection, whereas type A and B did not. Among *fimA* types, the survival rates of the larvae injected with rFimA type C were remarkably decreased, while the survival rates of the larvae injected with rFimA type A and type B were greater than 50%. Clindamycin treatment inhibited the growth of type C strains in a dose-dependent manner, resulting in an increased rate of silkworm survival. Finally, type C rFimA-speciﬁc antiserum prolonged the survival of silkworm larvae stimulated by infection with *P. gulae* type C strain or injection of rFimA type C protein.

**Conclusion**: These results suggested that type C fimbriae have high potential for enhancement of bacterial pathogenesis, and that both clindamycin and anti-type C rFimA-specific antibodies are potent inhibitors of type C fimbriae-induced toxicity. This is the first report to establish a silkworm infection model using *P. gulae* for toxicity assessment.

## Introduction

*Porphyromonas gulae*, a Gram-negative black-pigmented anaerobe, is the predominant species of *Porphyromonas* found in the periodontal pocket in dogs [1]. Characterization of *P. gulae* has shown it to be a major factor related to periodontal disease in dogs [2]. Recent studies have reported that *P. gulae* was detected in the gingiva and other periodontal tissues from healthy owners of companion animals [3,4], and that it invaded human gingival epithelial cells [5]. *P. gulae* was also shown to inhibit cell migration in mammalian cells and induce production of inﬂammatory cytokines, including interleukin (IL)-1β, IL-6, IL-8, and tumor necrosis factor–α [3,6,7]. These findings suggest that *P. gulae* may be involved in the development of periodontitis.

Many important virulence factors, including fimbriae, proteases, endotoxins, and capsules, are involved in bacterial pathogenesis and infectious disease [8,9]. Fimbrillin protein (FimA), the major subunit of fimbriae, is displayed on the cell surface, where it can play roles in several important functions, including the capacity to facilitate adhesion, colonization, biofilm formation, signal transduction, enzymatic degradation, and motility [5,10,11]. For several pathogens, including *Streptococcus parasanguis, P. gingivalis* and *Salmonella enterica*, FimA has been reported to impact bacterial pathogenesis [12–14]. The fimbriae of *P. gingivalis* have been classified into six genotypes (types I-V and Ib) based on the nucleotides sequences [15]. Several phenomena of the host–pathogen relationship are reportedly dependent on the *fimA* genotype, namely periodontal health status, bacterial adhesion to and invasion of host cells, and inflammatory induction [11,15–17], suggesting that clonal variations in ﬁmbriae may be related to *P. gingivalis* virulence [16,17]. *P. gulae* is well known as the animal biotype of the human periodontal pathogen *P. gingivalis* [18]. *P. gulae* fimbriae are hair-like appendages on the bacterial surface and are considered to be a critical virulence factor, mediating host tissues adherence and invasion [3]. Fimbriae from both *P. gulae* and *P. gingivalis* are classified into three major genotypes: A, B and C, with type C showing high virulence for periodontal disease, followed by B and then A [3,4,7]. *P. gulae* with type C fimbriae also exhibit greater levels of virulence in animal and human host cells compared with the other genotypes [4,7,19,20]. These reports suggest that the virulence of *P. gulae* may depend, to some extent, on the clonal diversity of fimbriae. However, the interaction between *P. gulae* infection and toxicity in an *in vivo* model has yet to be investigated.

The present study is the first to describe the silkworm *P. gula*e infection model, in which we examined optimal conditions for the assessment of virulence, and analyzed antibiotics sensitivity and response to anti-rFimA-speciﬁc antibodies.

## Materials and methods

### Bacterial culture conditions

*P. gulae* strains ATCC 51700 (*fimA* type A) [7], D040 (*fimA* type B) [7], and D049 (*fimA* type C) [3] were used in this study. Each strain was isolated from an oral swab specimen from a dog and confirmed to be *P. gulae* using a molecular biological method described previously [3]. *P. gulae* strains were cultured in Trypticase soy broth (Becton, Dickinson and Co., Franklin Lakes, NJ, USA) supplemented with yeast extract (1 mg/ml), hemin (5 µg/ml), and menadione (1 µg/ml), as described previously [5].

*Escherichia coli* DH5α (Nippon Gene, Tokyo, Japan) and *E. coli* BL21 (Nippon Gene) were used as host strains for transformation of plasmid DNA. *E. coli* strains were grown in Luria-Bertani (LB; 1% tryptone, 0.5% yeast extract, 0.5% NaCl) medium; LB agar was prepared by the addition of 1.5% agar. When necessary, kanamycin sodium (100 µg/ml) was added to the medium.

### Construction of plasmids containing recombinant fimbrillin genes

*P. gulae* genomic DNA was isolated as previously described [3] and used as a template for amplifying the entire *fimA* gene by PCR. The primers used for PCR were constructed from the sequences of the *fimA* genotypes [3] as follows: type A forward primers, 5′-GCG CGC GAA TTC GAG ATG AAA AAG ACT AAG-3′ and reverse primers, 5′-GCG CGG TTT AAG CTT TGA TTA CCA AGT AGC-3′; type B forward primers, 5′-GCG AAC GGA TCC AAG ATG AAA AAG ACT AAG-3′ and reverse primers, 5′-CGC TCT CTC GAG AGC TGA TTA CCA AAT-3; type C forward primers, 5′- ATC GAT ATC CAC TTT TAA AAC AAA AAA GAG-3′ and reverse primers 5′-TTT AGT CGT TTG ACG GGT CGA TTA CCA AGT-3′. Each amplified DNA was cloned into pGEM-T Easy Vector (Promega, Madison, WI), then digested with the appropriate restriction enzyme for ligation into the pET 42a (+) glutathione S-transferase (GST) gene fusion-protein expression vector (Novagen, Madison, WI, USA). The ligated vector was transformed into *E. coli* DH5α and colonies were selected on LB agar plates containing 50 µg of ampicillin/ml. Plasmid DNA was obtained using the Wizard® Plus SV Minipreps DNA Purification System (Promega). Each recombinant plasmid was transformed into *E. coli* BL21 (DE3) (Nippon Gene) and the colonies were selected as described above.

### Generation and purification of recombinant fimbrillin (rFimA)

**Figure 1.**
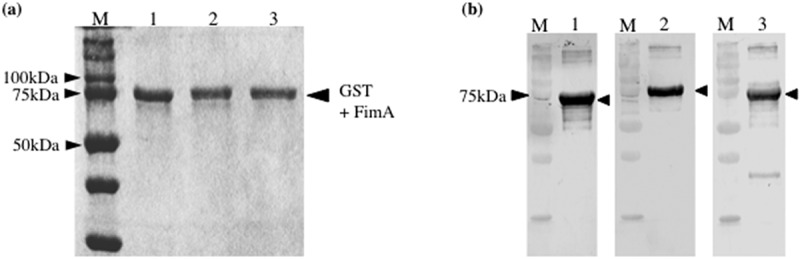
**Expression of recombinant FimA in *Escherichia coli* BL21**. (a) Coomassie blue staining of purified GST-rFimA fusion proteins separated by SDS-PAGE. Lane 1, rFimA type A, Lane 2, rFimA type B, Lane 3, rFimA type C. (b) Western blot analysis using each anti-rFimA antibody. Lane 1, rFimA type A; Lane 2, rFimA type B; Lane 3, rFimA type C. M: molecular marker

Production of each rFimA was induced with isopropyl-b-D-thiogalactopyranoside (IPTG), and the proteins were purified using a modification of a method described previously [21]. Briefly, an overnight culture (10 ml) of each transformed BL21 strain was used to inoculate 500 ml fresh LB broth containing 50 μg ampicillin/ml. The cultures were incubated at 37°C with vigorous shaking until the optical density at 600 nm reached 0.6, and then IPTG was added to a final concentration of 1 mM. After an additional 3 h of incubation to induce GST-rFimA fusion proteins, the cells were harvested by centrifugation at 2000 × g for 15 min at 4°C, and washed three times with phosphate-buffered saline (PBS) buffer. The cell pellets were suspended in the same buffer and ultrasonicated on ice. Supernatants were obtained by centrifugation and purified using a glutathione Sepharose™4B column (GE Healthcare, Fairfield, CT, USA). The purified GST-rFimA fusion proteins were subjected to sodium dodecyl sulfate-polyacrylamide gel electrophoresis (SDS-PAGE) and stained with Coomassie blue (Figure 1a). The purified proteins were dialyzed with MilliQ and freeze-dried.

### Generation of anti-rFimA antisera

Rabbit anti-rFimA antibodies were generated using a method described previously [21]. Purified rFimA protein was mixed with a block copolymer adjuvant (Titer-Max Gold; CytRx Co., Atlanta, GA, USA) and injected intramuscularly into adult New Zealand white rabbits weighing 1–1.5 kg (Oriental Yeast Co. Ltd., Tokyo, Japan) twice with a 14-day interval. One week after the second injection, blood was drawn, and antisera were collected and stored at −20°C. The antisera were separated by SDS-PAGE, transferred to a polyvinylidene fluoride membrane, and probed with anti-GST antibody to confirm the GST-rFimA antibody titers (Figure 1b).

### Antibiotic sensitivity of P. gulae

We used the following antibiotics: from Sigma (St. Louis, MO, USA), gentamicin and metronidazole; from Wako (Osaka, Japan), ampicillin and clindamycin. Freshly cultured *P. gula*e in the exponential phase of growth were employed so that antibiotics reactions could be observed for 24 h. Bacterial growth was measured on an SH-1000 Lab microplate reader (Corona Electric, Ibaraki, Japan) at 595 nm.

### Silkworm larvae model with P. gulae and rFimA

The silkworm infection experiments were performed as described previously [22]. Briefly, silkworms were raised from fertilized eggs to four-instar larvae in our laboratory. The hatched four-instar larvae were fed antibiotic-free food (SilkMate 2S, Nosan Corporation, Kanagawa, Japan) for 1 day. *P. gulae* suspensions (1 × 10^7^ or 5 × 10^7^ colony-forming units [CFU] in 50 μl PBS) and rFimA protein (5 µg in 50 μl) were injected into the silkworm hemolymph through the dorsal surface of the silkworms (n = 10) using a 26-gauge needle (Terumo, Tokyo, Japan). After injection, larvae were incubated at 37°C and their survival rate was measured every 24 h for 240 h. Several different concentrations of rFimA protein were preliminarily examined to determine the concentration used in this study (Supplementary Figure 1) with 5 µg determined as the appropriate concentration for comparing protein efficacy.

### Treatment with clindamycin and anti-FimA-specific antibodies

A *P. gulae* suspension (5 × 10^7^ CFU in 50 μl PBS) or rFimA protein (5 µg in 50 μl) was injected into the hemolymph of the silkworms (n = 10) using a 26-gauge needle (Terumo), and immediately followed by the injection of clindamycin and anti-rFimA specific antisera. The survival rate was assessed every 24 h for 240 h after injection.

### Statistical analyses

Statistical analyses were performed using the computational software packages StatView software (version 5.0, SAS Institute Inc., Cary, NC, USA). Student *t*-test was utilized to compare the effect of each antibiotic. Survival rates of the silkworm larvae in each group were evaluated with a Kaplan-Meier plot, which was analyzed by a log-rank test. A *P* value of <0.05 was considered to be statistically significant.

## Results

### Generation and purification of rFimA

We generated recombinant versions of all three *fimA* genotypes of *P. gulae* to determine the virulence of each. The molecular sizes of the rFimA proteins were all approximately 75 kDa (Figure 1a). We also generated antisera against the rFimA proteins and used western blotting to show that each antiserum reacted with its corresponding purified rFimA (Figure 1b). In addition, an enzyme linked immunosorbent assay (ELISA) was performed to analyze the results of each anti-rFimA assay (Supplementary Figure 2). Those findings demonstrated no cross-reactions and that specific results were obtained.

**Figure 2.**
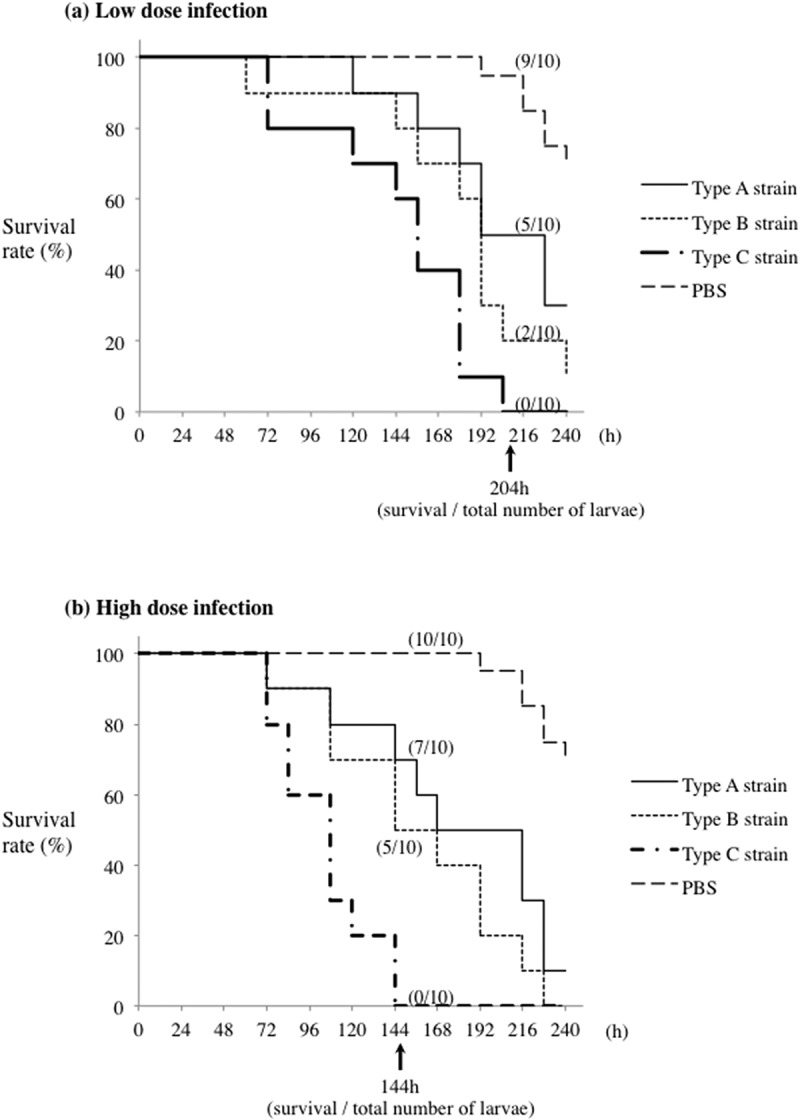
**Virulence of *P. gulae* infection in silkworm larvae**. larvae were injected with 10 ml suspension of *P. gulae*at. low dose (A; 1 × 10^7^ CFU) or high dose (B; 5 × 10^7^ CFU) and incubated at 37°C. The survival rate was recorded at the time points indicated. PBS was used as a negative control. Data are representative of three independent experiments

### P. gulae fimbriae involved in survival rate of silkworm larvae

**Figure 3.**
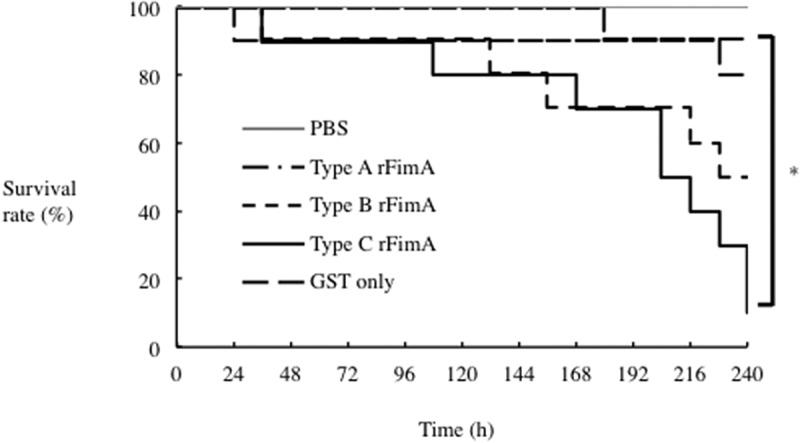
**Effect of *P. gulae* rFimA protein injection on silkworm larvae**. A total of 50 µl (5 µg) of each rFimA protein was injected into larvae and incubated at 37°C. The survival rate was recorded at the time points indicated. PBS was used as a negative control. Data are representative of three independent experiments. Survival rates in the silkworm larvae in each group were evaluated with a Kaplan–Meier plot, which was analyzed by a log-rank test (*P < 0.001)

Silkworm larvae have been characterized as an animal model of human infection with pathogenic bacteria [23]. Thus, we established and examined a *P. gulae* infection model using silkworm larvae. Figure 2 shows that, although 30% of the control larvae had died at 204 h under treatment with PBS, all of the larvae died within 204 h following infection with *P. gulae* D049 (type C) at the low dose (1 × 10^7^ CFU); by contrast, infections with *P. gulae* ATCC51700 (type A) and *P. gulae* D040 (type B) at the same dose were less virulent (Figure 2a). High-dose infection (5 × 10^7^ CFU) of *P. gulae* was found to eliminate the entire silkworm population at 144 h (Figure 2b). By comparison, at 228 h after a high-dose infection with types A and B, 10% of the larvae infected with *P. gulae* type A were still alive, while all of the larvae infected with *P. gulae* type B had died (Figure 2b). When larvae were injected with purified rFimA proteins, by the end of the experiment (240 h), the survival rates of those injected with all the rFimA proteins were lower as compared to those injected with GST only as well as the PBS control. Also, most (~90%) of the larvae injected with rFimA type C died, whereas the survival rate of larvae injected with rFimA type A or B exceeded 50% (Figure 3). We considered that this effect was caused by immune response and not cell disruption by a massive dose of recombinant proteins, since the survival rate of larvae injected with GST was only higher as compared to that received all recombinant proteins. These findings suggested that strains possessing type C fimbriae are more virulent in silkworm larvae than strains possessing type A or type B fimbriae.

### Effects of antibiotics treatment on P. gulae infection

**Figure 4.**
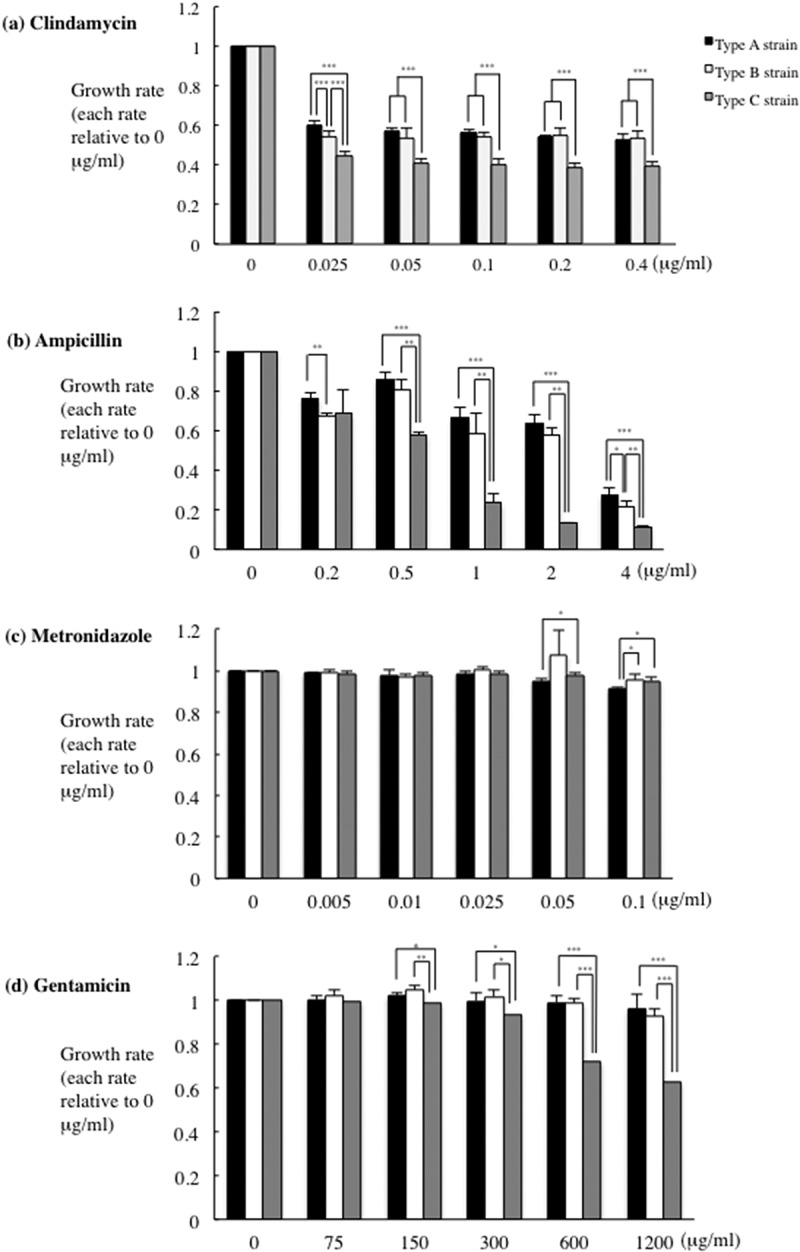
**Inhibitory effects of antibiotics on the growth of *P. gulae***. *P. gulae* (5 × 10^7^ CFU) was cultured in trypticase soy broth TSB and an antibiotic at the concentrations indicated for 24 h. after incubation, bacterial growth was measured using a microplate reader. Data are expressed as the relative ratio of treated/untreated cultures and represent means ± SD from three independent experiments analyzed with a *t*-test. *P < 0.05; **P < 0.01; ***P < 0.001

A recent investigation demonstrated the minimum inhibitory concentrations of antibiotics against *Porphyromonas* spp. and *Fusobacterium* spp. isolated from dogs [24], which was used to estimate the antimicrobial susceptibility of the examined *P. gulae* strains (Figure 4).

Clindamycin appeared to be the most effective against *P. gulae*, even at low doses (Figure 4a); by contrast, high dose of ampicillin, metronidazole and gentamicin were required to show similar levels of bacterial growth inhibition (Figure 4b-d). Previously reported findings indicated that metronidazole and gentamicin may offer a potentially useful therapeutic option for the treatment of *Porphyromonas* and *Prevotella* spp. infections [25]. Together with the present findings, it is suggested that clindamycin may be more effective against *P. gulae* strains as compared to ampicillin.

**Figure 5.**
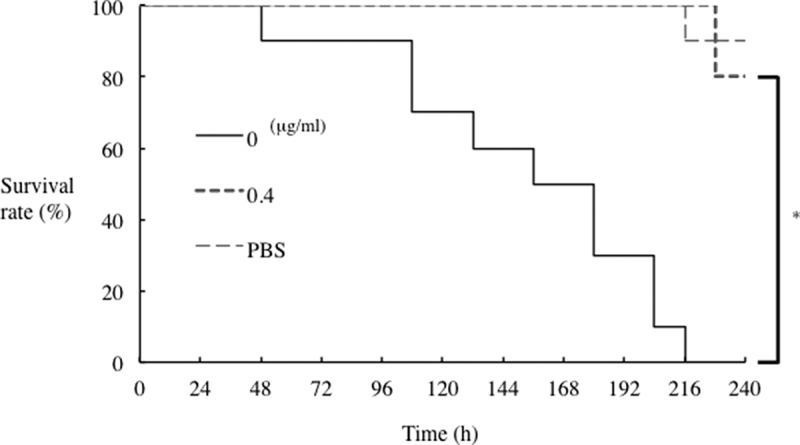
**Effect of clindamycin on *P. gulae* D049 (type C) infection of silkworm larvae**. Larvae (n = 10) were injected with a 50-µl suspension of *P. gulae* (5 × 10^7^ CFU) and a 50-μl clindamycin solution (0.4 μg/ml) and incubated at 37°C. The survival was recorded for the time indicated. PBS was used as a negative control. Data are representative of three independent experiments. Survival rates in the silkworm larvae in each group were evaluated with a Kaplan-Meier plot, which was analyzed by a log-rank test. *P < 0.001

**Figure 6.**
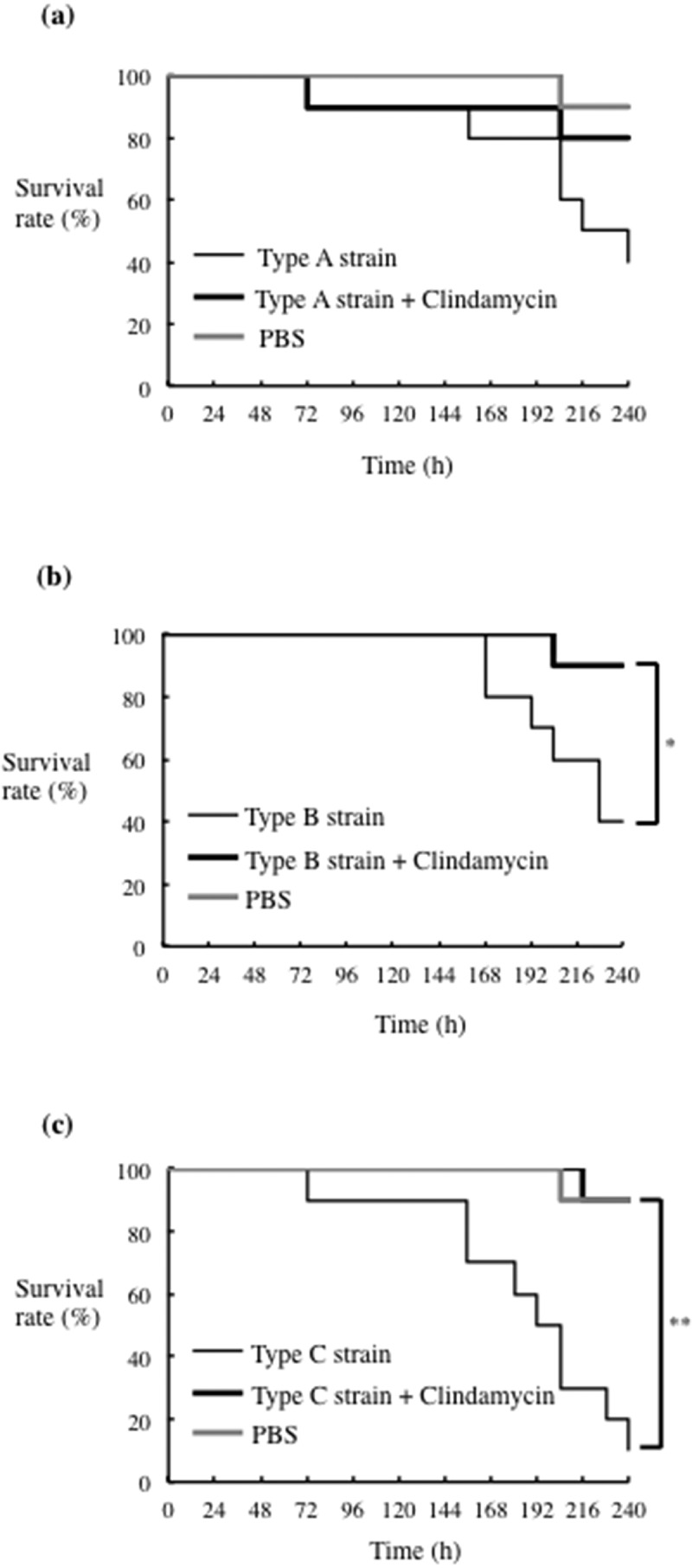
**Effect of clindamycin on infection of silkworm larvae with different *P. gulae* strains**. Larvae (n = 10) were injected with a 50-μl of *P. gulae* suspensions (5 × 10^7^ CFU) (a) strain ATCC51700 (type A), (b) strain D040 (type B), or (c) strain D049 (type C), and a 50-μl solution of 0.4 μg/ml clindamycin, and incubated at 37°C. The survival rate was recorded for the time points indicated. PBS was used as a negative control. Data are representative of three independent experiments. Survival rates in the silkworm larvae in each group were evaluated with a Kaplan-Meier plot, which was analyzed by a log-rank test. *P < 0.05; **P < 0.01

Inhibition of *P. gulae* by clindamycin led us to predict that clindamycin would improve survival of silkworm larvae infected with *P. gulae*. Initially, the inhibitory effects of a range of clindamycin concentrations on survival rates of larvae infected with *P. gulae* with type C fimbriae were examined and those results made it clear that clindamycin was effective against that infection (Figure 5). The survival rates of infected larvae improved in a concentration-dependent manner (Supplementary Figure 3). When we compared the effects of clindamycin on survival rates of infection with strains with all three fimbriae types, we found that the effect on type A infection was negligible, whereas survival of larvae infected with type B and type C strains was significantly improved (Figure 6). These results suggested that, of the four antibiotics tested, clindamycin may be the most effective against *P. gulae*-mediated infectious disease.

### Anti-rFimA antibodies improved the survival rate of silkworm larvae infection with P. gulae

**Figure 7.**
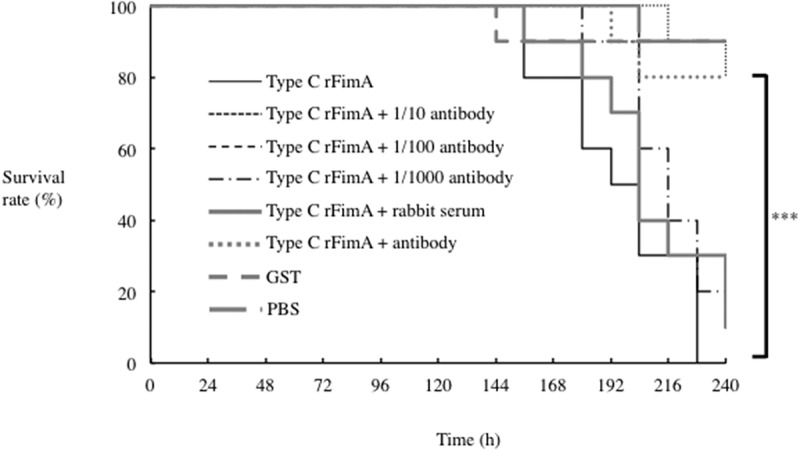
**Effects of rFimA type C antisera on survival of silkworm larvae injected with rFimA proteins**. Silkworm larvae (n = 10) were injected with 50 μl of each rFimA protein (5 μg each of type A, B and C) and anti-rFimA type C antisera, and incubated at 37°C. The survival rate was recorded for the time points indicated. Data are representative of three independent experiments. Survival rates in the silkworm larvae in each group were evaluated with a Kaplan-Meier plot, which was analyzed by a log-rank test. *P < 0.05; **P < 0.01

**Figure 8.**
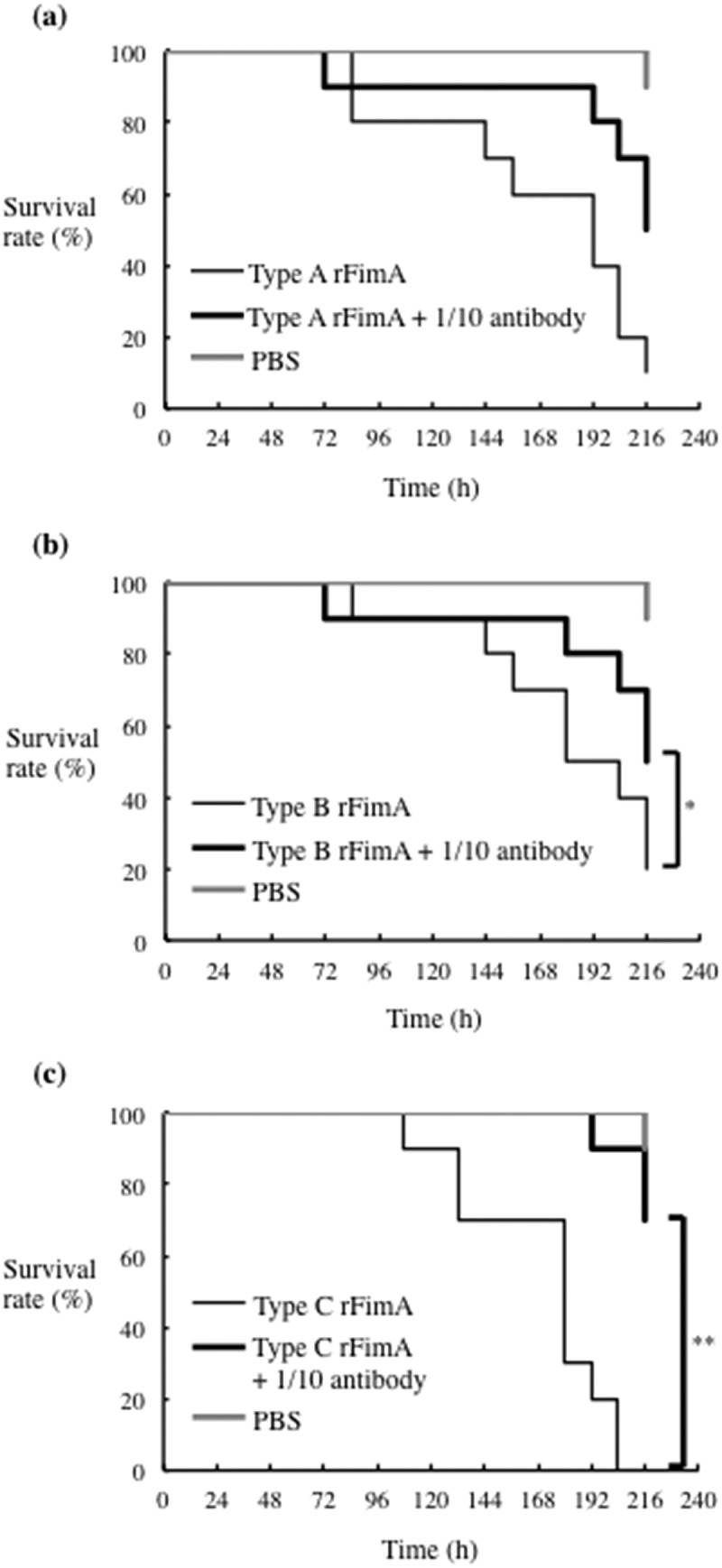
**Effects of antisera raised against each rFimA protein type on silkworm larvae injected with the corresponding rFimA protein**. Larvae (n = 10) were injected with 5 μg in 50 μl of type A (a), type B (b) or type C (c) rFimA protein, with without the corresponding anti-rFimA antisera (indicated concentrations), and incubated at 37°C. The survival rate was recorded for the time points indicated. PBS was used as a negative control. Data are representative of three independent experiments. Survival rates in the silkworm larvae in each group were evaluated with a Kaplan-Meier plot, which was analyzed by a log-rank test. *P < 0.05; **P < 0.01

**Figure 9.**
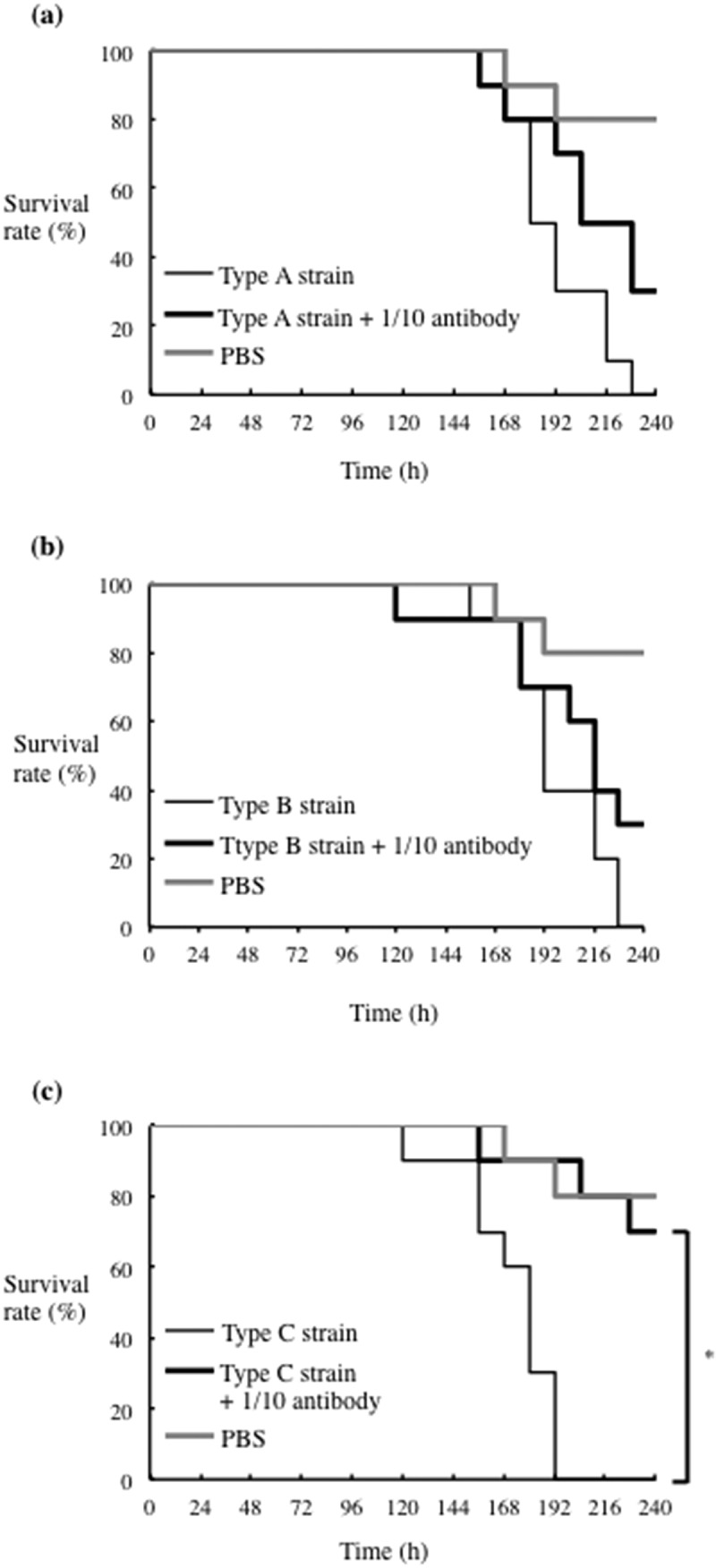
**Effect of antisera raised against each rFimA protein type on silkworm larvae infected with *P. gulae* strains**. Larvae (n = 10) were injected with a 50 μl suspension of *P. gulae* (5 × 10^7^ CFU) type A strain ATCC51700 (a), type B strain D040 (b), or type C strain (c), and were incubated at 37°C. The survival rate was recorded at the time points indicated. PBS was used as a negative control. Data are representative of three independent experiments. Survival rates in the silkworm larvae in each group were evaluated with a Kaplan-Meier plot, which was analyzed by a log-rank test. *P < 0.001

The virulence of bacterial fimbriae has been shown to be diminished by the use of anti-FimA antibodies [11,26]. Thus, we investigated the effect of antibodies generated against *P. gulae* rFimA proteins in the silkworm infection model. First, we examined a range of concentrations of type C rFimA antisera (1 to 1:1000 dilution) for their effects on the survival of injection of rFimA proteins. The most effective level of protection occurred at a 1:10 dilution (Figure 7), which was utilized in all subsequent experiments. Next, we examined antisera against rFimA type B and rFimA type C, and found that both improved the survival rates of larvae stimulated by the corresponding rFimA protein (Figure 8). When we tested each antiserum for protection of silkworms against infection with each *P. gulae* strain type, we found that type C and type B antibodies each conferred a survival advantage, while the effects of type A antisera were negligible (Figure 9).

## Discussion

Experiments in mammalian animal models, such as cats, ferrets, dogs, monkeys, mice and rats, have been used to assess pathogenicity, host susceptibility and virulence of microbial pathogens [27–31]. Microorganisms are also useful as biological agents to induce the inflammatory response and reveal mechanisms of pathogenesis in the rodent mice and rats [30,32]. However, the use of non-human primate research raises many issues related to cost, ethics, equipment, space, facilities, animal welfare and the potential for zoonosis [30,33]. Accordingly, recent studies have shown that non-vertebrate animal models, including *Caenorhabditis elegans*, Drosophila, silkworms and zebrafish, offer many advantages that make them versatile for research on bacterial pathogenesis and toxicity, and the assessment of antibiotics for infectious disease [23,34–36].

Silkworm larvae have also been useful for the characterization of bacterial pathogenicity, and the pharmacokinetics, pharmacodynamics and toxicology of drugs [37]. Silkworm larvae are quite commercially available, easily manipulated by human hands, and large enough to be injected by the same types of syringes used for human medical purposes [37]. Furthermore, the silkworm has an innate immune system akin to that in mammals, but lacks an acquired immune system [38]. Acquired resistance to pathogenic infection in silkworms, which is similar to a booster reaction, contributes to the enhancement of the immune response [39]. Silkworm muscle contraction was previously found to be strongly associated with the innate immune system of silkworms following stimulant administration [40]. Interestingly, *E. coli* LPS was found to not cause silkworm muscle contraction even at high doses [41], whereas highly purified LPS activated immune gene expression [42], suggesting that the recombinant protein expressed by *E. coli* could be useful for experiments with this model. Collectively, these reports indicate a high potential for a silkworm model as a replacement for animal experiments.

In the present study, silkworm larvae were established as a model for *P. gulae* infection, and used to assess the virulence of different strains (Figures 2 and Figure 3). The survival rate of silkworm larvae infected with *P. gulae* type C strain was lower compared with type A and type B strains (Figure 2), and injection of larvae with rFimA type C protein also dramatically lowered the survival rate (Figure 3). Our findings are consistent with several previous reports, showing virulence of *P. gula*e with type C fimbriae in mice and cultured host-cell models (5, 19, 20). The pathogenicity of *P. gingivalis* with respect to adherence to and invasion of epithelial cells depends on FimA type specificity (13). Our results suggested that the virulence of type C fimbriae is higher than that of other FimA types, and close relationships between each specific *fimA* genotypes and its pathogenesis were shown.

Results presented previously indicated that limitations of lethality–based studies of virulence are related to either the microbe or host variables [43]. Several pathogens, such as *Bacillus anthracis, Vibrio parahaemolyticus*, and *E. coli*, have been reported to show lethality in animal models [44–46]. In addition, there is a possibility that a *P. gingivalis* virulence factor, including fimbriae, could result in decreased survival of mice [17]. Moreover, *P. gingivalis* has been shown to have the ability to invade human host cells, degrade cellular focal adhesion components, and induce apoptosis in a *fimA*-types-dependent manner [16,47,48]. Recent studies have noted that *P. gulae* organisms express several different virulence factors, including fimbriae, LPS, and trypsin-like proteases [3,7,49,50]. *P. gulae* LPS was found to induce inflammatory responses in human gingival epithelial cells, while cell viability was not affected [49]. On the other hand, *P. gulae* proteases have been reported to degrade host tissue components related to growth and proliferation, resulting in promotion of morphological changes [50]. Also, *P. gulae* organisms with type C fimbriae have been shown to have greater levels of virulence towards mouse and human oral epithelial cells [3,5]. However, *in vitro* lethal and/or toxicity models of *P. gulae* fimbriae related to bacterial infection have yet to be investigated. In the present study, *P. gulae* type C strain with recombinant FimA type C caused a dramatic decrease in survival rate (Figures 2, Figure 3; Supplemental Figure 1). The present findings obtained with an *in vivo* model stimulated with fimbriae are considered to reflect those obtained with *in vitro* models of *P. gulae* as well as *P. gingivalis*. Together, these results suggest that type C fimbriae are a significant virulence factor of *P. gulae*.

The silkworm infection model is also useful for evaluating antibiotics and novel therapeutically effective agents for pathogenic bacterial infection [23,37]. The silkworm model is already being used to evaluate *Staphylococcus aureus* cell-wall proteins and regulatory proteins as virulence factors, as well as antibiotics for human infections with bacterial pathogens, including *S. aureus, Pseudomonas aeruginosa* and *Vibrio cholerae* [23,51]. Clindamycin is a semisynthetic derivative of the lincosamide class of compounds used clinically to treat gram-positive bacterial infections [52]. In this study, clindamycin demonstrated effective antimicrobial action against *P. gulae* growth, while metronidazole showed negligible effects (Figure 4). In addition, the effect of clindamycin was lower concentration as compared with its ampicillin (Figure 4). Further, administration of clindamycin during infection of silkworm larvae reduced the growth of *P. gulae* (Figure 6). Furthermore, the survival time of silkworm larvae injected with *P. gulae* type C was prolonged by the administration of clindamycin (Figure 6). Interestingly, clindamycin was more effective against *P. gulae* with type C fimbriae than strains with type A or B fimbriae, indicating that functional inhibition of type C fimbriae may be an important therapeutic target for prevention of *P. gulae*-mediated infectious disease. A previous report noted that anti-FimA antibodies improved arthritis mediated by microorganisms [26]. In addition, adhesion of type II FimA in *P. gingivalis* fimbriae to epithelial cells and bacterial invasion of cells were almost completely inhibited by anti-type II FimA antibodies [11], demonstrating the efficiency of anti-FimA antibodies for the reduction of *P. gingivalis* pathogenesis [11]. In the present study, the administration of rFimA antibodies prolonged the survival times of larvae infected with *P. gulae* (Figures 7–9). Together, these results suggested that our silkworm *P. gulae* infection model is suitable for the assessment of therapeutic effectiveness of antimicrobial reagents, such as antibiotics and antibodies.

In conclusion, we established a silkworm *P. gulae* infection model that could be useful for the assessment of candidate therapeutic agents. Furthermore, the prolongation of silkworm survival by administration of anti-rFimA antibodies could lead to the development of new therapies for periodontal diseases. Clindamycin is a candidate with therapeutic potential for anti-host-toxicity mediated by *P. gulae* colonization in periodontal disease.

## Supplementary Material

Supplemental Material

## References

[1] Kato Y, Shirai M, Murakami M, et al. Molecular detection of human periodontal pathogens in oral swab specimens from dogs in Japan. J Vet Dent. 2011;28(2):84–13. 21916371 10.1177/089875641102800204

[2] Hamada N, Takahashi Y, Watanabe K, et al. Molecular and antigenic similarities of the fimbrial major components between *Porphyromonas gulae* and *P. gingivalis*. Vet Microbiol. 2008;128(1–2):108–117. 17977673 10.1016/j.vetmic.2007.09.014

[3] Yamasaki Y, Nomura R, Nakano K, et al. Distribution and molecular characterization of *Porphyromonas gulae* carrying a new *fimA* genotype. Vet Microbiol. 2012a;161(1–2):196–205. 22877518 10.1016/j.vetmic.2012.07.026

[4] Iwashita N, Nomura R, Shirai M, et al. Identification and molecular characterization of *Porphyromonas gulae fimA* types among cat isolates. Vet Microbiol. 2019;229:100–109. 30642584 10.1016/j.vetmic.2018.12.018

[5] Inaba H, Nomura R, Kato Y, et al. Adhesion and invasion of gingival epithelial cells by *Porphyromonas gulae*. PLoS One. 2019;14(3):e0213309. 30870452 10.1371/journal.pone.0213309 PMC6417775

[6] Holden JA, O’Brien-Simpson NM, Lenzo JC, et al. *Porphyromonas gulae* activates unprimed and gamma interferon-primed macrophages via the pattern recognition receptors toll-like receptor 2 (TLR2), TLR4, and NOD2. Infect Immun. 2017;85(9):e00282–17. 28630066 10.1128/IAI.00282-17 PMC5563586

[7] Nomura R, Shirai M, Kato Y, et al. Diversity of Fimbrillin among *Porphyromonas gulae* clinical isolates from Japanese Dogs. J Vet Med Sci. 2012;74(7):885–891. 22382732 10.1292/jvms.11-0564

[8] Holt SC, Ebersole JL. *Porphyromonas gingivalis, Treponema denticola*, and *Tannerella forsythia*: the ‘red complex’, a prototype polybacterial pathogenic consortium in periodontitis. Periodontol. 2000;2005(38):72–122. 10.1111/j.1600-0757.2005.00113.x 15853938

[9] Casadevall A, Pirofski LA. Virulence factors and their mechanisms of action: the view from a damage-response framework. J Water Health. 2009;1(7 Suppl):S2–S18. 10.2166/wh.2009.036 19717929

[10] Klemm P, Schembri MA. Fimbrial surface display systems in bacteria: from vaccines to random libraries. Microbiology. 2000;146(12):3025–3032. 11101660 10.1099/00221287-146-12-3025

[11] Nakagawa I, Amano A, Kuboniwa M, et al. Functional differences among FimA variants of *Porphyromonas gingivalis* and their effects on adhesion to and invasion of human epithelial cells. Infect Immun. 2002;70(1):277–285. 11748193 10.1128/IAI.70.1.277-285.2002 PMC127611

[12] Burnette-Curley D, Wells V, Viscount H, et al. FimA, a major virulence factor associated with *Streptococcus parasanguis* endocarditis.. Infect Immun. 1995;63(12):4669–4674. 7591121 10.1128/iai.63.12.4669-4674.1995 PMC173670

[13] Amano A. Molecular interaction of *Porphyromonas gingivalis* with host cells: implication for the microbial pathogenesis of periodontal disease. J Periodontol. 2003;74(1):90–96. 12593602 10.1902/jop.2003.74.1.90

[14] Musa HH, Zhang WJ, Duan XL, et al. The molecular adjuvant mC3d enhances the immunogenicity of FimA from type I fimbriae of *Salmonella enterica* serovar Enteritidis. J Microbiol Immunol Infect. 2014;47(1):57–62. 23352331 10.1016/j.jmii.2012.11.004

[15] Amano A, Nakagawa I, Okahashi N, et al. Variations of *Porphyromonas gingivalis* fimbriae in relation to microbial pathogenesis. J Periodontal Res. 2004;39(2):136–142. 15009522 10.1111/j.1600-0765.2004.00719.x

[16] Inaba H, Kawai S, Kato T, et al. Association between epithelial cell death and invasion by microspheres conjugated to *Porphyromonas gingivalis* vesicles with different types of fimbriae. Infect Immun. 2006;74(1):734–739. 16369031 10.1128/IAI.74.1.734-739.2006 PMC1346634

[17] Inaba H, Nakano K, Kato T, et al. Heterogenic virulence and related factors among clinical isolates of *Porphyromonas gingivalis* with type II fimbriae. Oral Microbiol Immunol. 2008;23(1):29–35. 18173795 10.1111/j.1399-302X.2007.00386.x

[18] Fournier D, Mouton C, Lapierre P, et al. *Porphyromonas gulae* sp. nov., an anaerobic, gram-negative coccobacillus from the gingival sulcus of various animal hosts.. Int J Syst Evol Microbiol. 2001;51(3):1179–1189. 11411686 10.1099/00207713-51-3-1179

[19] Yamasaki Y, Nomura R, Nakano K, et al. Distribution of periodontopathic bacterial species in dogs and their owners. Arch Oral Biol. 2012b;57(9):1183–1188. 22417880 10.1016/j.archoralbio.2012.02.015

[20] Nomura R, Inaba H, Yasuda H, et al. Inhibition of *Porphyromonas gulae* and periodontal disease in dogs by a combination of clindamycin and interferon alpha. Sci Rep. 2020;10(1):3113. 32080231 10.1038/s41598-020-59730-9 PMC7033253

[21] Matsumoto‐Nakano M, Tsuji M, Amano A, et al. Molecular interactions of alanine-rich and proline-rich regions of cell surface protein antigen c in *Streptococcus mutans*. Oral Microbiol Immunol. 2008;23(4):265–270. 18582324 10.1111/j.1399-302X.2007.00421.x

[22] Kaito C, Kurokawa K, Matsumoto Y, et al. Silkworm pathogenic bacteria infection model for identification of novel virulence genes. Mol Microbiol. 2005;56(4):934–944. 15853881 10.1111/j.1365-2958.2005.04596.x

[23] Kaito C, Akimitsu N, Watanabe H, et al. Silkworm larvae as an animal model of bacterial infection pathogenic to humans. Microb Pathog. 2002;32(4):183–190. 12079408 10.1006/mpat.2002.0494

[24] Senhorinho GN, Nakano V, Liu C, et al. Occurrence and antimicrobial susceptibility of *Porphyromonas* spp. and *Fusobacterium* spp. in dogs with and without periodontitis. Anaerobe. 2012;18(4):381–385. 22609780 10.1016/j.anaerobe.2012.04.008

[25] Stephan B, Greife HA, Pridmore A, et al. Activity of pradofloxacin against *porphyromonas* and *prevotella* spp. implicated in periodontal disease in dogs: susceptibility test data from a European multicenter study. Antimicrob Agents Chemother. 2008;52(6):2149–2155. 18411326 10.1128/AAC.00019-08 PMC2415797

[26] Jeong SH, Nam Y, Jung H, et al. Author Correction: interrupting oral infection of *Porphyromonas gingivalis* with anti-FimA antibody attenuates bacterial dissemination to the arthritic joint and improves experimental arthritis. Exp Mol Med. 2018;50(8):113. 30158563 10.1038/s12276-018-0140-z PMC6115335

[27] Kuhnert P, Boerlin P, Frey J. Target genes for virulence assessment of *Escherichia coli* isolates from water, food and the environment. FEMS Microbiol Rev. 2000;24(1):107–117. 10640601 10.1111/j.1574-6976.2000.tb00535.x

[28] Liu L, Zhao H, Zhang Y, et al. Neonatal rhesus monkey is a potential animal model for studying pathogenesis of EV71 infection. Virology. 2011;412(1):91–100. 21262515 10.1016/j.virol.2010.12.058

[29] Hoogland ICM, Houbolt C, Van Westerloo DJ, et al. Systemic inflammation and microglial activation: systematic review of animal experiments. J Neuroinflammation. 2015;12(1):114. 26048578 10.1186/s12974-015-0332-6 PMC4470063

[30] Jiminez JA, Uwiera TC, Inglis DG, et al. Animal models to study acute and chronic intestinal inflammation in mammals. Gut Pathog. 2015;7:29. 26561503 10.1186/s13099-015-0076-y PMC4641401

[31] Shi J, Wen Z, Zhong G, et al. Susceptibility of ferrets, cats, dogs, and other domesticated animals to SARS-coronavirus 2. Science. 2020;2020(368):1016–1020. 10.1126/science.abb7015 PMC7164390 32269068

[32] Hokamura K, Inaba H, Nakano K, et al. Molecular analysis of aortic intimal hyperplasia caused by *Porphyromonas gingivalis* infection in mice with endothelial damage. J Periodontal Res. 2010;45(3):337–344. 19909399 10.1111/j.1600-0765.2009.01242.x

[33] Normand R, Du W, Briller M, et al. Found in translateon: a machine model for mouse-to-human interence. Nat Methods. 2018;15(12):1067–1073. 30478323 10.1038/s41592-018-0214-9 PMC12396630

[34] Barnoy S, Gancz H, Zhu Y, et al. The *Galleria Mellonella* larvae as an *in vivo* model for evaluation of *Shigella* virulence. Gut Microbes. 2017;8(4):335–350. 28277944 10.1080/19490976.2017.1293225 PMC5570432

[35] Bastos PAD, De Costa JP, Vitorino R. A glimpse into the modulation of posttranslational modifications of human-colonizing bacteria. J Proteomics. 2017;152:254–275. 27888141 10.1016/j.jprot.2016.11.005

[36] Mitchell CL, Latuszek CE, Vogel KR, et al. α-amanitin resistance in Drosophila melanogaster, A genome-wide association approach. PLoS One. 2017;12(2):e0173162. 28241077 10.1371/journal.pone.0173162 PMC5328632

[37] Panthee S, Paudel A, Hamamoto H, et al. Advantages of the silkworm as an animal model for developing novel antimicrobial agents. Front Microbiol. 2017;8:373. 28326075 10.3389/fmicb.2017.00373 PMC5339274

[38] Kurokawa K, Kaito C, Sekimizu K. Two‐component signaling in the virulence of *Staphylococcus aureus*: a silkworm larvae‐pathogenic agent infection model of virulence. Methods Enzymol. 2007;422:233–244. 17628142 10.1016/S0076-6879(06)22011-1

[39] Miyashita A, Kizaki H, Kawasaki K, et al. Primed immune responses to gram-negative peptidoglycans confer infection resistance in silkworms. J Biol Chem. 2014;289(20):14412–14421. 24706746 10.1074/jbc.M113.525139 PMC4022907

[40] Nishida S, Ishii M, Nishimiya Y, et al. Lactobacillus paraplantarum 11–1 isolated from rice bran pickles activated innate immunity and improved survival in a silkworm bacterial infection model. Front Microbiol. 2017;8:346. 28373863 10.3389/fmicb.2017.00436 PMC5357627

[41] Dhital S, Hamamoto H, Urai M, et al. Purification of innate immunostimulant from green tea using a silkworm muscle contraction assay. Drug Discov Ther. 2011;5(1):18–25. 22466092 10.5582/ddt.v5.1.18

[42] Tanaka H, Sagisaka A, Fujita K, et al. Lipopolysaccharide elicits expression of immune‐related genes in the silkworm, *Bombyx mori*. Insect Mol Biol. 2009;18(1):71–75. 19196348 10.1111/j.1365-2583.2009.00851.x

[43] Casadevall A, Pirofski L. Host‐pathogen interactions: the attributes of virulence. J Infect Dis. 2001;184(3):337–344. 11443560 10.1086/322044

[44] Firoved AM, Miller GF, Moayeri M, et al. Bacillus anthracis edema toxin causes extensive tissue lesions and rapid lethality in mice. Am J Pathol. 2005;167(5):1309–1320. 16251415 10.1016/S0002-9440(10)61218-7 PMC1603774

[45] Johnson JR, Clermont O, Menard M, et al. Experimental mouse lethality of *Escherichia coli*isolates, in relation to accessory traits, phylogenetic group, and ecological source. J Infect Dis. 2006;194(8):1141–1150. 16991090 10.1086/507305

[46] Hiyoshi H, Kodama T, Iida T, et al. Contribution of Vibrio parahaemolyticus virulence factors to cytotoxicity, enterotoxicity, and lethality in mice. IAI. 2010;78(4):1772–1780. 10.1128/IAI.01051-09 PMC2849405 20086084

[47] Nakagawa I, Inaba H, Yamamura T, et al. Invasion of epithelial cells and proteolysis of cellular focal adhesion components by distinct types of *Porphyromonas gingivalis* fimbriae. IAI. 2006;74(7):3773–3782. 10.1128/IAI.01902-05 PMC1489697 16790749

[48] Kato T, Uzawa A, Ishihara K. Inhibitory effect of galectin-3 on the cytokine-inducing activity of periodontopathic *Aggregatibacter actinomycetemcomitans* endotoxin in splenocytes derived from mice. FEMS Immunol Med Microbiol. 2009;57(1):40–45. 19619243 10.1111/j.1574-695X.2009.00577.x

[49] Inaba H, Yoshida S, Nomura R, et al. *Porphyromonas gulae* lipopolysaccharide elicits inflammatory responses through toll‐like receptor 2 and 4 in human gingivalis epithelial cells. CM. 2020;22:e13254. 10.1111/cmi.13254 32827217

[50] Urmi AS, Inaba H, Nomura R, et al. Roles of *Porphyromonas gulae* proteases in bacterial and host cell biology. Cell Microbiol. 2021;24:e13312. 10.1111/cmi.13312 33486854

[51] Miyazaki S, Natsumoto Y, Sekimizu K, et al. Evaluation of *Staphylococcus aureus* virulence factors using a silkworm model. FEMS Microbiol Lett. 2012;326(2):116–124. 22092964 10.1111/j.1574-6968.2011.02439.x

[52] Dunkle JA, Xiong L, Mankin AS, et al. [2010]. Structures of the *Escherichia coli* ribosome with antibiotics bound near the peptidyl transferase center explain spectra of drug action. Proc Natl Acad Sci USA. 2010; 107(40):17152–17157. 20876128 10.1073/pnas.1007988107 PMC2951456

