# Supplementary material for: Efficacy of FimA antibody and clindamycin in silkworm larvae stimulated with Porphyromonas gulae
Source: J Oral Microbiol. 2021 Apr 25;13(1):1914499. doi: 10.1080/20002297.2021.1914499 (PMC8079003; doi:10.1080/20002297.2021.1914499)
Supplement: Supplemental Material [file ZJOM_A_1914499_SM7513.zip › Supplementary files/supplementray figure caption.docx]

**Supplementary Figure 1 Effects of various concentrations of *P. gulae* rFimA protein injected into silkworm larvae.** A total of 50 µl (5 µg) of each rFimA protein was injected into larvae and incubation was performed at 37°C. Survival rate was recorded at the time points indicated. PBS was used as a negative control. Data are representative of three independent experiments. Survival rates in the silkworm larvae in each group were evaluated with a Kaplan-Meier plot, which was analyzed by a log-rank test (**P*<0.001).

**Supplementary Figure 2 ELISA analysis using each anti rFimA antibody.** (a) Type A rFimA, (b) Type B rFimA, (c) Type C rFimA.

**Supplementary Figure 3 Effects of various concentrations of clindamycin on silkworm larvae infected with *P. gulae* D049 (type C)*.*** Larvae (n = 10) were injected with a 50-µl suspension of *P. gulae* (5 × 10^7^ CFU) and a 50-μl clindamycin solution (concentrations indicated) and incubated at 37°C. Survival rate was recorded at the times indicated. PBS was used as a negative control. Data are representative of three independent experiments. Survival rates in the silkworm larvae in each group were evaluated with a Kaplan-Meier plot, which was analyzed by a log-rank test. **P* < 0.001.
