# Supplementary figures and images for: Efficacy of FimA antibody and clindamycin in silkworm larvae stimulated with Porphyromonas gulae
Source: J Oral Microbiol. 2021 Apr 25;13(1):1914499. doi: 10.1080/20002297.2021.1914499 (PMC8079003; doi:10.1080/20002297.2021.1914499)

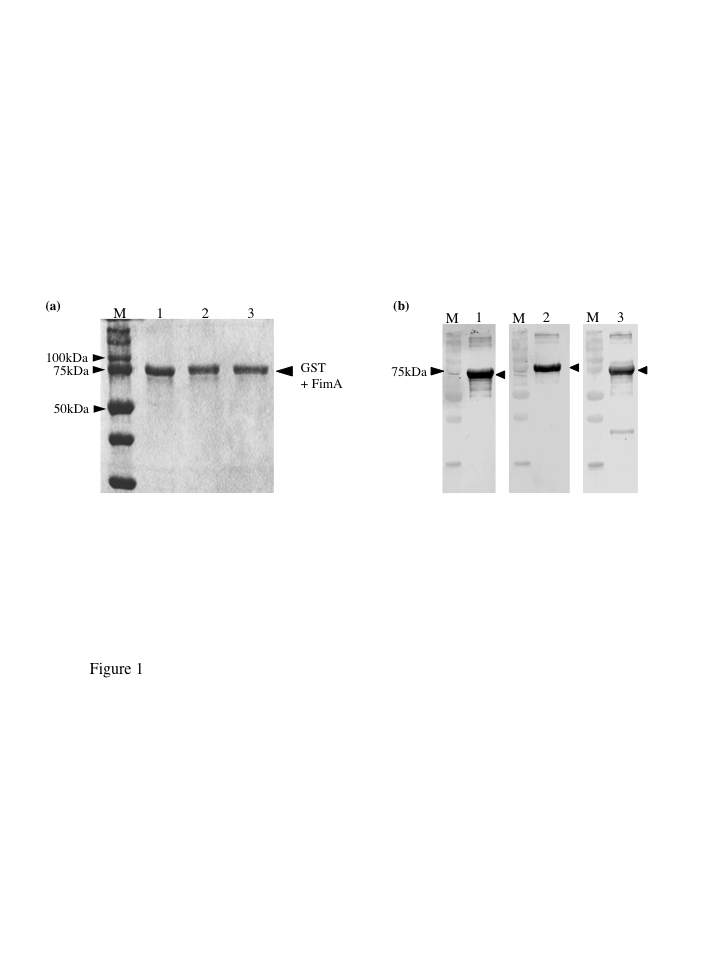

Supplement: Supplemental Material [file ZJOM_A_1914499_SM7513.zip › Supplementary files/Supple_Figure_1.tiff]

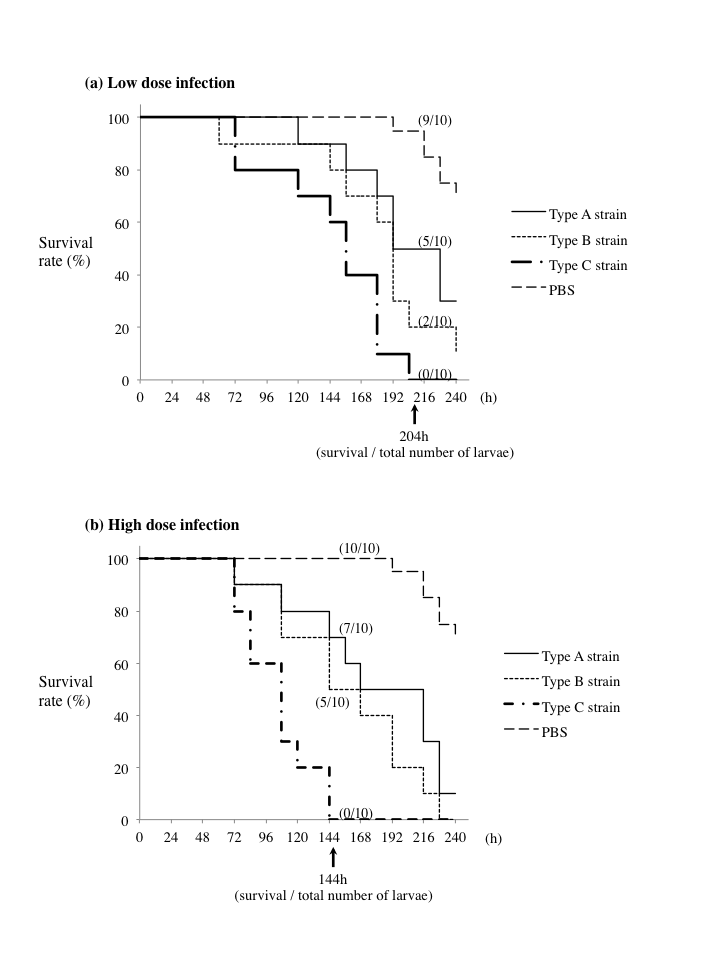

Supplement: Supplemental Material [file ZJOM_A_1914499_SM7513.zip › Supplementary files/Supple_Figure_2.tiff]

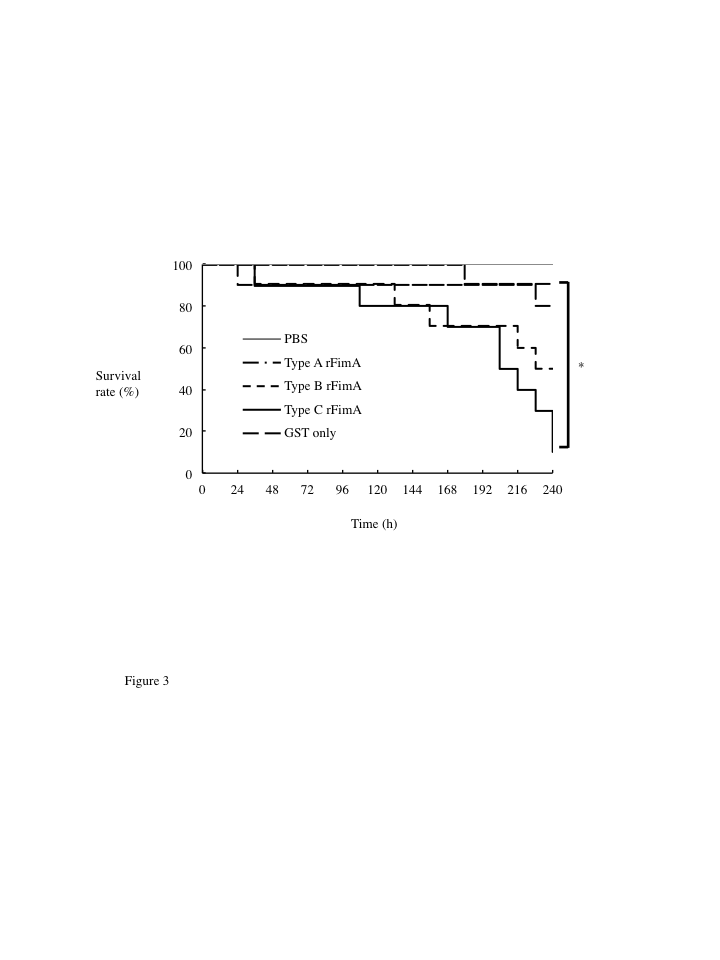

Supplement: Supplemental Material [file ZJOM_A_1914499_SM7513.zip › Supplementary files/Supple_Figure_3.tiff]
